# Delivering WOOP for Dementia Caregivers to Groups via Technology: A Feasibility Study

**DOI:** 10.1093/geroni/igaf122.672

**Published:** 2025-12-31

**Authors:** Donna Fedus, Emily Mroz, Joan Monin, Thi Vu, Lauren Lewis

**Affiliations:** Borrow My Glasses, Madison, Connecticut, United States; Emory University, Atlanta, Georgia, United States; Yale, Yale, Connecticut, United States; Yale University, New Haven, Connecticut, United States; Borrow My Glasses, Madison, Connecticut, United States

## Abstract

WOOP for Dementia Caregivers (WFDC) is an online health technology program and platform that teaches the evidence-based strategy Wish>Outcome>Obstacle>Plan (WOOP) to caregivers of people living with dementia. WOOP helps individuals engage in self-care by identifying ways to overcome internal obstacles within their control to fulfill important, challenging wishes. This session describes an NIA-funded Small Business Technology Transfer (STTR) study, where a small business (woman-owned gerontology education and evaluation company Borrow My Glasses) collaborated with scholars at research institutions (Yale University and other academic colleagues) and community members to develop WFDC. This session reports qualitative findings from focus group discussions with 24 caregiver-participants who engaged with WFDC. Caregivers learned and practiced WOOP during three online group sessions and received support via a password-protected WFDC website. Analysis of six focus group discussions revealed factors that motivated successful implementation of WFDC: individual motivators like caregivers honoring their commitment to themselves and prioritizing wellbeing; interpersonal motivators like the benefits of group practice and unique facilitator attributes; and programmatic motivators like the fact that WOOP is a simple approachable tool, and the program provides a gentle supportive culture balanced with parameters to maintain progress. Participants also described barriers to successful implementation, including individual barriers of balancing WOOP with other responsibilities and caregiver readiness for support; interpersonal barriers of limited opportunities to maintain relationships with group members and interpersonal tensions; and programmatic barriers of inconsistencies implementing study materials and scheduling constraints. These findings suggest ways to refine WFDC and benefit others developing technology-based programs for caregivers.

